# Assessment of exposure to ionizing radiation in Chernobyl tree frogs (*Hyla orientalis*)

**DOI:** 10.1038/s41598-021-00125-9

**Published:** 2021-10-15

**Authors:** Pablo Burraco, Clément Car, Jean-Marc Bonzom, Germán Orizaola

**Affiliations:** 1 Animal Ecology, Department of Ecology and Genetics, Evolutionary Biology Centre, Uppsala University, 75236 Uppsala, Sweden; 2 Institute of Biodiversity, Animal Health and Comparative Medicine, College of Medical, Veterinary and Life Sciences, University of Glasgow, Glasgow, G12 8QQ UK; 3 Research Laboratory on the Effects of Radionuclides on Ecosystems (LECO), Institute for Radioprotection and Nuclear Safety (IRSN), PSE-ENV/SRTE/LECO, 13115 Cadarache, Saint Paul Lez Durance France; 4 IMIB-Biodiversity Research Institute (Univ. Oviedo-CSIC-Princip. Asturias), University of Oviedo, 33600 Mieres-Asturias, Spain; 5 Zoology Unit, Department of Biology of Organisms and Systems, University of Oviedo, 33071 Oviedo-Asturias, Spain

**Keywords:** Herpetology, Environmental sciences, Pollution remediation

## Abstract

Ionizing radiation can damage organic molecules, causing detrimental effects on human and wildlife health. The accident at the Chernobyl nuclear power plant (1986) represents the largest release of radioactive material to the environment. An accurate estimation of the current exposure to radiation in wildlife, often reduced to ambient dose rate assessments, is crucial to understand the long-term impact of radiation on living organisms. Here, we present an evaluation of the sources and variation of current exposure to radiation in breeding Eastern tree frogs (*Hyla orientalis*) males living in the Chernobyl Exclusion Zone. Total absorbed dose rates in *H. orientalis* were highly variable, although generally below widely used thresholds considered harmful for animal health. Internal exposure was the main source of absorbed dose rate (81% on average), with ^90^Sr being the main contributor (78% of total dose rate, on average). These results highlight the importance of assessing both internal and external exposure levels in order to perform a robust evaluation of the exposure to radiation in wildlife. Further studies incorporating life-history, ecological, and evolutionary traits are needed to fully evaluate the effects that these exposure levels can have in amphibians and other taxa inhabiting radio-contaminated environments.

## Introduction

Living organisms are constantly exposed to ionizing radiation. Cosmic rays, together with naturally occurring radioactive materials, generate low-level radiation known as background radiation^1^. Ionizing radiation has the capacity to damage organic molecules, including DNA, either directly by breaking DNA strains or through the generation of free radicals^2^. The main concern about the impact of ionizing radiation in wildlife is not generated by background radiation, but by the release of radioactive material to the environment due to human actions. These actions include nuclear weapons tests, mining of radioactive material, and accidents in nuclear facilities. The accidents in the nuclear power plants of Chernobyl (Ukraine, 1986) and Fukushima (Japan, 2011) represent the largest accidental releases of ionizing radiation to the environment in human history. In order to reduce human exposure to radiation after these accidents, human settlement and normal activity were banned within certain areas, known as *Exclusion Zones*. In the absence of humans, wildlife may represent key study systems in which to examine the effects of the long-term exposure to ionizing radiation. Although the effects on ecosystems of the acute exposure to ionizing radiation were severe right after the Chernobyl accident^3^, there are still many uncertainties about the impact that chronic exposure to lower levels of ionizing radiation can have on wildlife^4–7^. An accurate assessment of the exposure to ionizing radiation is needed to properly evaluate its consequences on the health of wild populations and across taxa.

The International Commission for Radiological Protection (ICRP) determined reference levels of radiation exposure called Derived Consideration Reference Levels (DCRLs), defined as a band of dose rate within which there is likely to be some chance of deleterious effects of ionizing radiation occurring to individuals^8^. Different bands have been determined for a set of Reference Animals and Plants (RAPs^8^). However, RAPs are restricted to a few animal and plant taxa, and DCRLs are defined mostly based on theoretical predictions or short-term laboratory procedures, thus they do not include the complexities of ecosystems, where organisms are often exposed to a wide array of fluctuating conditions and stressors (see e.g.^9^). Since RAPs are just reference organisms, defined at family level and sometimes purely theoretical for entire animal or plant groups (e.g. “eusocial bee” defining all types of insects^8^), they do not include either basic differences in species life styles, physiology, or morphology. Other thresholds levels have been proposed for organisms and ecosystems by different organizations (e.g. ERICA, FASSET, Environment Agency UK, Environment Canada; see summary in^10^), with threshold levels above the lower values of ICRP ranges, in most cases. More field studies, conducted in RAP, and non-RAP organisms and under ecologically relevant scenarios, are clearly needed to understand the variability of exposure levels in wildlife.

More than three decades have passed since the Chernobyl nuclear power plant accident, a time that approximately corresponds to the half-life (i.e. the time required for a 50% reduction of the initial levels at the time of the accident) of ^90^Sr and ^137^Cs, the two main radioisotopes currently present in the Chernobyl Exclusion Zone^11^. Radiation levels in Chernobyl Exclusion Zone are now several orders of magnitude lower than at the time of the accident, and they are generated by a different array of radioisotopes^12^. An accurate evaluation of current exposure to ionizing radiation in wildlife inhabiting the Chernobyl Exclusion Zone needs to consider the contribution of different radionuclides and radiation types (alpha, beta and gamma), and go beyond the use of portable dosimeters, which only estimate ambient dose rates, account only for gamma radiation, and do not distinguish between the contribution of different radioisotopes^11^. A detailed estimation of the current levels of exposure to ionizing radiation in wildlife living in radio-contaminated areas is crucial to assess the risk that radioactive substances can represent for these organisms, to provide a proper dosimetry context for understanding the effects (or lack of effects) of ionizing radiation in ecologically-realistic scenarios, and to estimate the accuracy of the proposed reference levels used in radiological assessment.

In this study, we examine the most important sources and the variation of the exposure to ionizing radiation in breeding Eastern tree frog (*Hyla orientalis*) males living within the Chernobyl Exclusion Zone. ICRP uses a theoretical frog as a reference for predicting radiosensitivity in amphibians, for which a band of 40–400 μGy/h was defined within which it is likely to start detecting deleterious effects^8^. The ERICA Tool, one of the most widely used software to assess radiological risk to terrestrial, freshwater and marine biota, used a default screening dose rate of 10 μGy/h for protecting organisms living in natural ecosystems^13^. We use these references thresholds since they are widely used by the radioecology community, and are also two of the most conservative ones, with critical levels normally below other proposed references^10^. Previous studies have reported a wide variation in the contribution of internal versus external exposure in amphibians, as well as differences in radioisotope contributions between species and areas (e.g.^14^). Here, we estimate absorbed dose rates in tree frogs collected during three consecutive breeding seasons (2016–2018) across a wide gradient of radioactive contamination within the Chernobyl Exclusion Zone. In order to have a precise estimation of the current exposure to ionizing radiation in wild tree frogs, we not only quantified ambient dose rates, but also external exposure to radiation (from soil and water) and internal exposure in adult breeding frogs by integrating the activity of both ^90^Sr in bones and ^137^Cs in muscles. We expected to find a high contribution of internal dose rates and ^90^Sr^14^, as well as high variability in absorbed dose rates across the Chernobyl Exclusion Zone. The understanding of the variability in radiation exposure in wild amphibians is critical for further evaluations of potential life-history and eco-evolutionary effects of radiation.

## Results

### Ambient dose rates across tree frog’s breeding habitats in the Chernobyl Exclusion Zone

**Figure 1:**
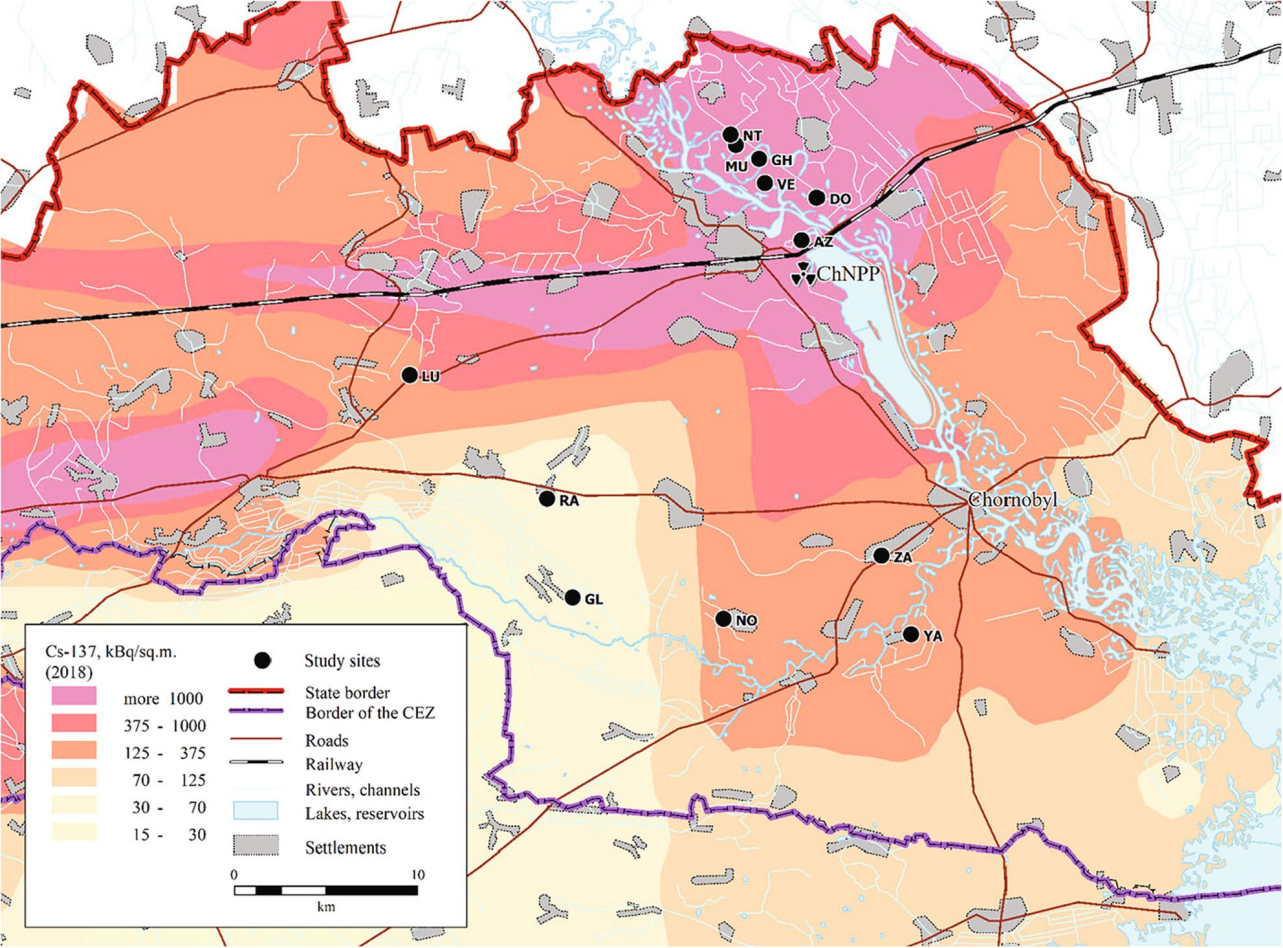
Map showing the localities where males of the Eastern tree frog (*Hyla orientalis*) were sampled. The abbreviations refer to the locality name. Vershina (VE), Azbuchin (AZ), Muravka (MU), Glyboke Hydro (GH), Northern Trace (NT), Dolzhikovo (DO), Lubianka (LU), Novosiolki (NO), Zalesie (ZA), Yampol (YA), Glinka (GL), and Razjezzheie (RA; see Table 1 for details). Map created with MapInfo Pro 17.0.3 (https://www.precisely.com/product/precisely-mapinfo), the underlying ^137^Cs soil data (decay corrected to spring 2018) is derived from the Atlas of Radioactive Contamination of Ukraine (http://radatlas.isgeo.com.ua/)^37^.

Ambient dose rates measured at the twelve *H. orientalis* breeding localities sampled within the Chernobyl Exclusion Zone ranged from 0.07 to 32.40 µSv/h (Table 1). Six localities had ambient dose rates above 1 µSv/h and are located in areas commonly considered as highly contaminated (> 1000 kBq/m^2^ of ^137^Cs in 2018; Fig. 1). Six additional localities had ambient dose rates below 0.3 µSv/h (< 375 kBq/m^2^ of ^137^Cs in 2018; Fig. 1).

**Table 1:** Geographic coordinates (latitude and longitude), and current levels of environmental radiation (i.e. ambient dose rate) of the Eastern tree frog (*Hyla orientalis*) breeding localities included in the study.

Locality	Code	GPS coordinates	Ambient dose rate (µSv/h)
Vershina	VE	51.4328, 30.0769	16.20
Azbuchin	AZ	51.4047, 30.1044	32.40*
Muravka	MU	51.4515, 30.0528	3.70
Glyboke Hydro	GH	51.4447, 30.0711	3.70
Northern Trace	NT	51.4567, 30.0486	2.51
Dolzhikovo	DO	51.4256, 30.1161	2.10**
Lubianka	LU	51.3388, 29.7976	0.27
Novosiolki	NO	51.2195, 30.0430	0.13
Zalesie	ZA	51.2506, 30.1667	0.12
Yampol	YA	51.2119, 30.1899	0.10
Glinka	GL	51.2300, 29.9250	0.10
Razjezzheie	RA	51.2786, 29.9050	0.07

*7.61 µSv/h in 2017 and 2018; **1.09 µSv/h in 2017, 1.50 µSv/h in 2018, differences due to small changes in sampling areas within each locality and year.

### Radioactivity concentration in tree frog’s bones and muscles

Among the 226 male Eastern tree frogs (*Hyla orientalis*) examined, 65 individuals had activity concentrations below detection levels for ^90^Sr (29%), and 35 individuals for ^137^Cs (15%). In individuals from localities with ambient dose rate > 1 μSv/h (the localities with most individuals presenting activity concentrations above detection levels), ^90^Sr activity in bones ranged from 4 to 1156 Bq/g (fresh mass), which represents 0.4 to 115 Bq/g of whole-body concentration (Supplementary data). Activity concentrations for ^137^Cs, measured in muscle tissue of individuals from localities with ambient dose rate > 1 μSv/h, ranged from 1.5 to 57 Bq/g (fresh mass), representing 0.09 to 39 Bq/g of whole-body concentration (Supplementary data). The contribution of ^90^Sr to the total activity concentration of frogs living in localities with ambient dose rate > 1 μSv/h was, overall, two-fold higher than that of ^137^Cs (66% ^90^Sr contribution *versus* 33% of ^137^Cs contribution, on average).

### Absorbed dose rates of *H. orientalis* within Chernobyl Exclusion Zone

**Figure 2:**
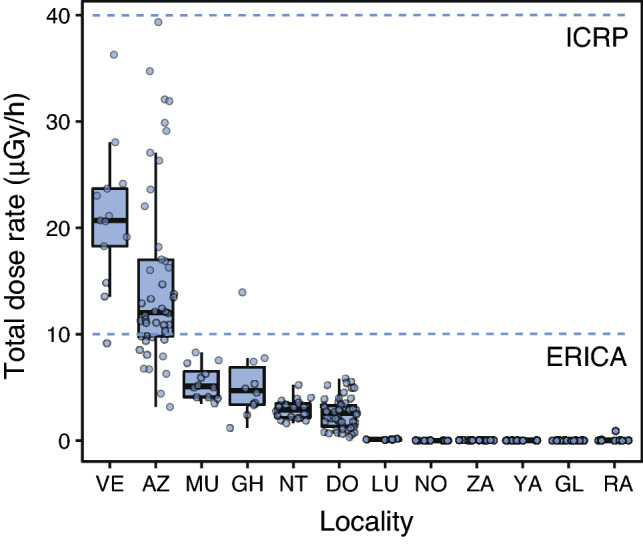
Total absorbed dose rates (μGy/h) of male breeding Eastern tree frogs (*Hyla orientalis*) living within Chernobyl Exclusion Zone. ICRP’s 40 μGy/h level for detecting damage on the reference frog, and ERICA’s 10 μGy/h screening level for protecting organisms within ecosystems are depicted with dotted lines. See Fig. 1 for correspondence of locality.

**Figure 3:**
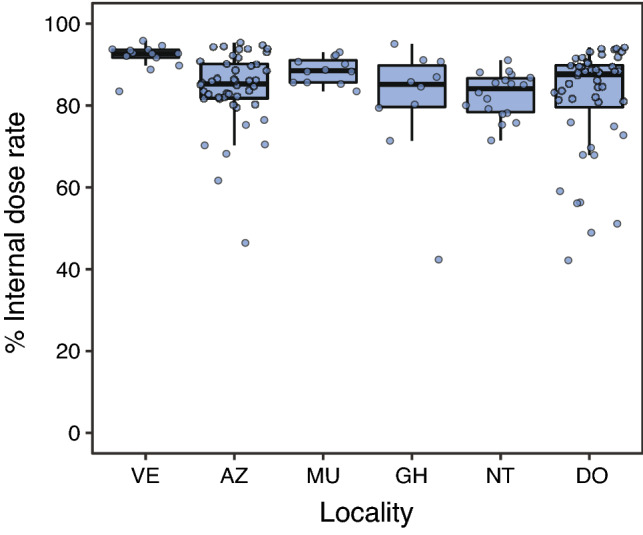
Contribution of internal dose rates (in percentage) to total individual dose rates absorbed by breeding Eastern tree frog (*Hyla orientalis*) males collected within the Chernobyl Exclusion Zone (only in individuals from localities with ambient dose rate > 1 μSv/h). See Fig. 1 for correspondence of locality.

**Figure 4:**
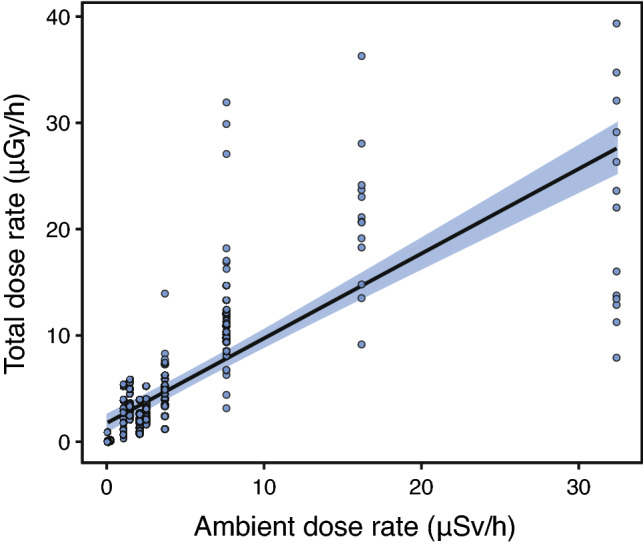
Correlation between ambient dose rates (in μSv/h) and total absorbed dose rates (in μGy/h) in breeding Eastern tree frog (*Hyla orientalis*) males living in the Chernobyl Exclusion Zone.

**Figure 5:**
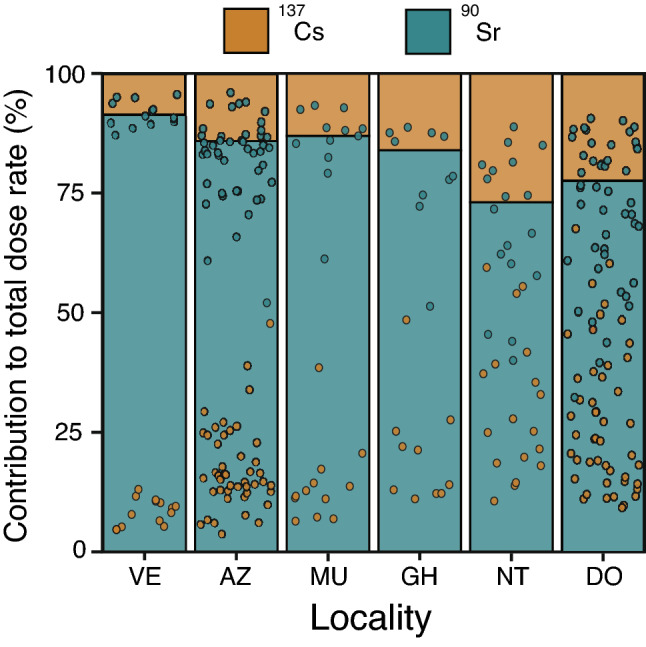
Contribution of ^90^Sr and ^137^CS (in percentage), to total dose rate absorbed by breeding Eastern tree frog (*Hyla orientalis*) males living in the Chernobyl Exclusion Zone (in individuals from localities with ambient dose rate > 1 μSv/h). Bars represent the locality average contribution for both isotopes, and points the contributions of each individual/isotope combination. See Fig. 1 for correspondence of locality.

Total weighted absorbed dose rates of *H. orientalis* males ranged between 0.01 and 39.35 μGy/h among individuals with activity rates above detection levels (Table 2, Fig. 2). Total absorbed dose rates varied substantially within and among localities: locality average values ranged from ca. 0 to 20 μGy/h (arithmetic mean; Table 2, Fig. 2). All sampled individuals had total absorbed dose rates below ICRP’s 40 μGy/h level for the reference frog^8^, whereas ca. 20% (n = 46) had rates above ERICA’s 10 μGy/h screening dose rate limit for protecting ecosystems^13^ (Fig. 2). Internal absorbed dose rates ranged between 0.01 and 37.49 μGy/h, whereas external dose rates ranged between ca. 0 and 2.0 μGy/h (Table 2; Fig. S1). Internal and external dose rates were highly positively correlated (conditional R^2^ = 0.93, marginal R^2^ = 0.91; Fig. S1). For individuals living in areas where ambient dose rate was > 1 μSv/h (i.e. with individual absorbed dose rates above minimal detectable activities, see “Methods”), the contribution of internal dose rate to the total individual absorbed dose rate was always higher than the contribution of the external dose rate (83% of contribution of the internal dose rate, on average; χ ^2^_(1,147)_ = 14.41, p < 0.001; Fig. 3). There was a highly significant and positive correlation between ambient dose rate and total individual absorbed dose rate (χ^2^_(1,226)_ = 15.21, p < 0.001, Estimate = 0.187; conditional R^2^ = 0.95; marginal R^2^ = 0.07; Fig. 4). The contribution of ^90^Sr represented, on average, 78% of the total absorbed dose rate of frogs living in localities with ambient dose rate > 1 μSv/h (Fig. 5), with a contribution of ^90^Sr to the internal dose rate six-fold higher than that of ^137^Cs (86% ^90^Sr contribution *versus* 14% of ^137^Cs contribution, on average, Fig. S2), and a 35% contribution of ^90^Sr to the external dose rate (Fig. S3, Supplementary data).

**Table 2:** Dose rates of breeding Eastern tree frog (*Hyla orientalis*) males captured within the Chernobyl Exclusion Zone (2016–2018).

Locality	Code	Sampled frogs (n)	Internal dose rate (μGy/h)	External dose rate (μGy/h)	Total dose rate (μGy/h)
Vershina	VE	13	19.45 (7.65–34.77)	1.51	20.96 (9.16–36.28)
Azbuchin	AZ	46	13.31 (1.47–37.49)	1.82 (1.69–2.00)	15.13 (3.16–39.35)
Muravka	MU	12	4.89 (2.92–7.72)	0.58	4.81 (3.50–8.29)
Glyboke Hydro	GH	10	4.72 (0.51–13.25)	0.69	5.41 (1.19–13.94)
Northern Trace	NT	18	2.46 (1.16–4.74)	0.46	2.93 (1.63–5.20)
Dolzhikovo	DO	49	2.25 (0–5.48)	0.33 (0.33–0.34)	2.58 (0.33–5.82)
Lubianka	LU	5	0.09 (0.05–0.14)	0.03	0.11 (0.10–0.17)
Novosiolki	NO	12	0 (MDA–0.02)	MDA	0.01 (MDA–0.02)
Zalesie	ZA	12	0.02 (MDA–0.04)	0.01	0.03 (0.02–0.05)
Yampol	YA	14	0 (MDA–0.01)	0.01	0.01 (0.01–0.02)
Glinka	GL	25	0.01 (MDA–0.05)	MDA	0.01 (MDA–0.05)
Razjezzheie	RA	10	0.11 (MDA–0.90)	MDA	0.11 (MDA–0.91)

Data presented as mean value (range). Only localities sampled more than 1 year had variation in external dose rates (see text). *MDA* minimum detectable activity.

## Discussion

Our study shows that radiation exposure, in breeding males of the Eastern tree frog (*Hyla orientalis*) inhabiting across a wide gradient of radioactive contamination in the Chernobyl Exclusion Zone, is highly variable and, overall, below international thresholds for detecting damage. Individual absorbed dose rates varied substantially both at the inter- and intra-locality level. Total absorbed dose rates in Chernobyl tree frogs during the breeding season are dominated by internal, rather than external radiation levels, and are primarily a consequence of the doses of ^90^Sr in bones. Finally, although total individual absorbed dose rates were positively correlated with ambient dose rates, our data indicate that using only ambient dose rates will result in a poor estimation of the exposure to radiation in our study species as this parameter does not reflect the inter-individual variation in absorbed radiation.

Despite our study comprehensibly sampled frogs within twelve different localities and across the gradient of radioactive contamination experienced by the species in the Chernobyl Exclusion Zone (Fig. 1), all individuals presented total absorbed dose rates below the ICRP threshold level of 40 μGy/h (and also below other standards set by multiple organizations, see^8,10^). When using the more conservative 10 μGy/h screening level suggested by ERICA for protecting ecosystems^13^, about 20% of the sampled *H. orientalis* were above this level, corresponding mainly to frogs collected in the two most radio-contaminated localities (AZ and VE, Fig. 2). Overall, these results suggest that three decades after the nuclear accident, exposure to radiation within the Chernobyl Exclusion Zone has dropped, in most cases, down below levels supposed to be damaging for these frogs during the breeding season (see^8^). Therefore, we can consider that negative effects of radiation are unlikely to be detected, except perhaps in the most radio-contaminated localities within Chernobyl (e.g. AZ and VE in our study, but see below). However, these values need to be interpreted regarding the ecological characteristics that underlie our sampling design, as dose rates were measured on breeding individuals that expend a large amount of time in shoreline environments. A higher exposure is likely expected for frogs buried in the ground or leaf litter during the hibernation period, and therefore the within-year and lifetime variation in radiation levels and its dependence on patterns of habitat occupancy deserves further exploration.

In our study, we examined ^90^Sr and ^137^Cs levels as sources of radiation for estimating internal and external dose rates. At present, ^90^Sr and ^137^Cs are the most abundant radioisotopes in the Chernobyl Exclusion Zone, whereas several radioisotopes with short half-life have already disappeared (e.g. ^131^I, ^132^Te, ^140^Ba^11^). However, other less abundant radionuclides such as ^241^Am, ^238^Pu, and ^239^Pu, are still present in the area and they may contribute to a fraction of the total absorbed dose rate accumulated by an organism^14^. Nonetheless, previous studies conducted in the Chernobyl Exclusion Zone have reported a minimal contribution of these low-abundant isotopes to total absorbed dose rates in amphibians^14^. Therefore, although our approach can slightly underestimate total dose rates in breeding tree frogs, we can consider that these differences should be minimal, and our absorbed dose rate estimates accurate.

Thresholds that determine radiation levels likely to cause damage are set without tests in ecological settings and can be slightly inaccurate (see discussion in e.g.^9^). In our study system, further studies will determine whether current radiation levels experienced by Chernobyl tree frogs can negatively impact their life-history and eco-evolutionary dynamics (see e.g.^15^). On this respect, there are important aspects that deserve further research. For example, we need to examine if the different life stages of *H. orientalis* can have a higher sensitivity to radiation than the ones predicted for the reference frog used by ICRP^8^, as a consequence of differences in shape, size or life history between tree frogs, and the parameters considered when defining the ICRP reference frog. As commented above, absorbed dose rate can also vary across seasons and during the life-time of an individual. Furthermore, other ecological stressors such as diseases, parasites, or droughts, combined with small but still relevant effects of ionizing radiation can contribute to generate imbalances in the physiology and life-history of tree frogs at radiation levels defined as safe^6^. Finally, effects of radiation currently observed can be a consequence of the impact of historical exposure to radiation, i.e. exposure to much higher radiation levels immediately after the accident^6,16^. Therefore, differences across the radiation gradient within the Chernobyl area, but also between contaminated and non-contaminated localities outside the Exclusion Zone, may be linked to transgenerational carry-over effects induced by radiation in the past (transferred either by genetic and/or epigenetic mechanisms^6^). Evaluating the relevance of these and other possible scenarios will improve our understanding on the effects that past and current exposure to ionizing radiation can have on wildlife.

Our results also reveal that radioecology studies using only ambient radiation levels will inaccurately estimate the exposure of organisms to radiation (see e.g. comments in^12,17^). We found a positive correlation between ambient dose rate and total individual absorbed dose rates in *H. orientalis*, suggesting that ambient radiation can be used to broadly define contamination areas for the species in Chernobyl. However, the high variation in total individual absorbed dose rates observed within each locality (i.e. for which ambient radiation is considered as a single value) indicates that using only ambient dose rates may lead to non-accurate estimates of the exposure to radiation experienced by each individual. Furthermore, previous studies on amphibians have revealed a large inter-specific variation in the contributions of internal and external dose rates. For example, internal dose rate represented ca. 40% of the total absorbed dose rate in moor frogs (*Rana arvalis*), ca. 50% in fire-bellied toads (*Bombina bombina*), and more than 70% in spadefoot toads (*Pelobates fuscus*), collected in the red forest area of Chernobyl Exclusion Zone^14^. In other areas, internal dose rate was reported to have a minimal contribution to the total absorbed dose rate of moor frogs (*Rana arvalis*), collected in ponds of central Sweden within areas contaminated from the Chernobyl fallout^18^. For other animal taxa, the contribution of internal dose rates can be as low as ca. 10% in bumblebees or ca. 20% in voles (*Microtus* spp.^14^). Our study reports some of the largest contributions of internal dose rates reported for wildlife (83% internal contribution to total absorbed dose rate^14^), and agrees with previous results in a similar species, the Japanese tree frog (*Hyla japonica*), examined in Fukushima and with internal dose rates contributing between 92–69% to the total absorbed dose rate^19^. Levels of ^90^Sr accumulated in the bones of *H. orientalis* contributed to most of the total individual absorbed dose rate (78%, on average), mostly due to its contribution to internal dose rate (86% on average, for frogs living in localities with ambient dose rate > 1 μSv/h). This also agrees with previous studies in Chernobyl reporting that ^90^Sr contributed between ca. 90% of the total dose rate in common toads (*Bufo bufo*) and spadefoot toads (*Pelobates fuscus*), and to a bit less than 60% in fire-bellied toads (*Bombina bombina*^14^). Overall, ^90^Sr is the main source of total absorbed dose rates among Chernobyl wildlife^14^. This is relevant since ^90^Sr accumulates in the bones, and therefore it can result in a relatively high tissue-specific dose rate. Additionally, absorbed dose rates in this study were estimated using IRSN-EDEN v3 software (see “Methods”) whereas most previous studies used the Erica Tool. Although both software generally provide comparable estimates^20,21^, this can lead to small differences between the estimates calculated by different studies. Apart from this technical point, our results confirm the need to conduct detailed evaluations of internal exposure (i.e. internal absorbed dose rates) in order to precisely determine exposure levels in wildlife^6,14,22^.

Overall, this study presents a detailed evaluation of the variability of current exposure to radiation in breeding Eastern tree frogs (*H. orientalis*) living within the Chernobyl Exclusion Zone. Our study reveals the need to estimate total individual absorbed dose rates (i.e. including both internal and external exposure), and to evaluate the most common radioisotopes in order to accurately assess wildlife exposure to radiation. Absorbed dose rates, in our study species, are below widely used ICRP bands and most other proposed thresholds^8,10^, whereas only 20% of the quantified dose rates were above ERICA screening levels for protecting ecosystems^13^. However, many uncertainties remain around the estimation of these thresholds (e.g.^9,10^), and therefore detailed studies incorporating life-history and eco-evolutionary variability are needed in order to properly evaluate the status of this species and other wildlife inhabiting Chernobyl.

## Methods

### Field sampling and laboratory procedures with *Hyla orientalis*

We used the Eastern tree frog (*Hyla orientalis*) as our study species. *Hyla orientalis* is a cryptic species of the European tree frog (*Hyla arborea*) group, distributed from the Caspian Sea to the Baltic Sea^23^. Females start to breed at 2–3 years of age^24^, which means that 10–15 generations have pass since the Chernobyl accident (1986). The species requires warm temperature for the start of the breeding season, which normally occurs in May–June in the study area. *H. orientalis* hibernates buried in the soil or under rocks, leaf litter or wood. Adults feed on a large diversity of small arthropods.

During three consecutive years (2016–2018), we collected adult males of *H. orientalis* actively calling during the breeding season in ponds located within the Chernobyl Exclusion Zone (Ukraine, Fig. 1). In total, we examined 226 *H. orientalis* males from twelve localities within the Chernobyl Exclusion Zone (Table 1; Fig. 1). Some localities were sampled more than one year (AZ, DO, and GL in Fig. 1, see Table 2) as part of different projects (e.g.^15^). We did not detect breeding activity of the species in other highly contaminated areas (e.g. Red Forest, which are usually areas with sandy soils and likely unsuitable, too dry, as good breeding habitats for the species), so our sampled localities are a good representation of the entire range of radioactive contamination experienced by the species in the Chernobyl Exclusion Zone. Frogs were captured during the night (from 10 pm to 1am), placed in plastic bags and transported to our field laboratory in Chernobyl. On the next morning, we recorded different morphological traits of each frog (snout-to-vent length, body depth and width) using a calliper to the nearest 1 mm, and we weighted each individual using a precision balance to the nearest 0.01 g. Morphometric measurements were used to define individual shapes in order to estimate individual absorbed dose rates (see below). Once morphometric measurements were recorded, we euthanized frogs by pithing without decapitation (as recommended by AVMA^25^), and tissue and bone samples were stored for radiological evaluation. The study followed ARRIVE (https://arriveguidelines.org) and AVMA guidelines^25^. All animals were collected, and experimental protocols conducted under permit and ethical approval of Ministry of Ecology and Natural Resources of Ukraine (licence No. 517, 21.04.2016). All methods were performed in accordance with the relevant ethical guidelines and regulations.

### Field estimation of radiation levels

At each locality, we estimated ambient dose rate using a radiometer MKS-AT6130 to measure both gamma dose rate (µSv/h) and the flux of beta particles (counts cm^−2^ min^−1^) in three environments: above water (measured at ca. 5 cm above the surface of water in places with 0.3–1.0 m depth), along the shoreline (defined as the water-land interface, usually with presence of vegetation), and in surrounding terrestrial environment, using five points at each environment type, covering the tree frog sampling area. In most cases, the shoreline values had lower variability, while the terrestrial and water values varied substantially. We assume that shoreline values are more indicative of the environment used by frogs during the breeding season (i.e. we captured frogs in shoreline vegetation), and therefore we used those values for dose assessment (Table 1).

### External exposure: deposits of ^90^Sr and ^137^Cs in the soil and water of the study localities

In order to estimate radioactive levels of the study localities, and its contribution to external dose rates, we used a spatial database derived from the integration of the airborne gamma survey and the results of soil sampling in earlier 1990s^26^. The final database represents a geo-positioned 100 × 100 m grid with values of total ^90^Sr and ^137^Cs deposits after the Chernobyl accident (in Bq/m^2^). To estimate ^90^Sr and ^137^Cs activity in soil for the sampling localities, we followed Gashchack et al*.*^27^, and calculated the geometric mean (n = 50 points) from these integrated databases over a 400 m radius area centred on the study pond (selected to cover the post-hibernation range of the species, see e.g.^28^), and activity estimates were decay-corrected to the time of the current study (spring 2016–2018; Fig. 1). In order to reconstruct soil activity concentration, we assume a value of 160 kg/m^2^ ^29^, for the top 10 cm of soil. We estimated activity in water (In Bq/L) using soil activities and distribution coefficients estimated for the Glubokoye lake^30^. A similar approach, and spatial database, has been previously used in studies of other animals with relatively large home ranges, when direct evaluation of soil or water samples was unfeasible (amphibians^31^; birds^32^; rodents^33^; bats^27^).

### Internal exposure: estimation of ^90^Sr activity concentration in bones

Relatively high ^90^Sr activity concentration is found in the bones of animals living in the Chernobyl Exclusion Zone^27,32,33^, which allows the application of standard beta spectrometry methods^34^. In our study, we sampled a femur bone of every frog that was thoroughly cleaned up from remains of soft tissues. Then, we dried the bone sample in order to estimate dry mass to the nearest 0.01 g. After this, we diluted the sample with concentrated HNO_3_ and H_2_O_2_. We evaporated the obtained solution to generate wet salts, followed by the addition of 1 M HNO_3_ to standardize the geometry. We used the final solution for beta-spectrometry, and recalculated the obtained data to the dry mass values of each sample. We used a β-spectrometer EXPRESS-01 with a thin-filmed (0.1 mm) plastic scintillator detector, with the software “Beta+” (developed by the Institute of Nuclear Research at the National Academy of Science of Ukraine). This method allows to measure ^90^Sr content in thick-layered samples with a comparable ^137^Cs content (^137^Cs/^90^Sr ratio not exceeding 30:1^34^). We processed the obtained experimental spectrum using correlations with the measured spectra from OISN-3 standard mixing sources (Applied Ecology Laboratory of Environmental Safety Centre, Odessa, Ukraine; e.g. ^90^Sr + ^90^Y, ^137^Cs and the ^90^Sr + ^90^Y, and ^137^Cs combinations), as well as from background. The minimum detectable activity (MDA) was 0.6 Bq per sample. The small mass of the bone samples and the relatively low contamination of frogs from some localities did not allow to estimate ^90^Sr activity concentration below MDA (Supplementary data).

### Internal exposure: estimation of ^137^Cs activity concentration in muscles

In order to estimate ^137^Cs levels, we sampled muscle tissue from frog legs. We measured the wet mass of the muscle sample to the nearest 0.01 g. Then, we diluted the muscle sample with concentrated HNO_3_ and H_2_O_2_. The obtained solution was evaporated to generate wet salts, followed by the addition of 1 M HNO_3_ to standardize the geometry. We used the final solution for gamma-spectrometry, and recalculated the obtained data to the wet mass values of each sample. We measured ^137^Cs activity concentrations on the muscle samples using a Canberra-Packard gamma-spectrometer with a high-purity germanium (HPGe) detector (GC 3019). A OISN-1 standard mixed source (^44^Ti/^137^Cs/^152^Eu; Applied Ecology Laboratory of Environmental Safety Centre, Odessa, Ukraine), including epoxy granules (< 1.0 mm) with 1 g cm^−3^ density, was used for calibration. The minimally detectable activity ranged from 0.1 to 0.3 Bq per sample depending on sample mass, counting time, and radioactivity of the original sample. The small mass of the muscle samples and the relatively low contamination of frogs from some localities did not allow to estimate ^137^Cs activity concentration below MDA (Supplementary data).

### Estimation of individual total absorbed dose rates

To estimate total individual dose rates (TDR, in µGy/h) absorbed by each frog during the breeding season, we first estimated whole-body activity of ^90^Sr and ^137^Cs by integrating radionuclide activity concentrations (see above) with body mass of each individual, and considering the relative mass of bones (10%) and muscles (69%)^35^, we did not made any further assumptions for the rest of frog body weight. We combined radionuclide activity concentrations in frogs, soil, and water with dose coefficients (in µGy/h per Bq per unit of mass). The use of dose coefficients allows transforming radionuclide activity (Bq/kg, Bq/L) into dose rate (μGy/h), and are specific for each radionuclide/organism/ecological scenario combination. Dose coefficients for *H. orientalis* were calculated for internal and external exposure by taking into consideration a theoretical ecologically scenario for the species during a whole breeding period as follows: 8 h/day spent on vegetation at > 50 cm above ground, 8 h/day on the ground, 7h30/day at the water surface, and 30 min/day at the sediment–water interface (soil depth: 10 cm; water depth: 100 cm; grass depth: 10 cm; for a similar approach, see^19^). We calculate absorbed doses using IRSN-EDEN v3 software (https://www.irsn.fr/EN/Research/Scientific-tools/Computer-codes/Pages/The-EDEN-computer-code-Elementary-Dose-Evaluation-for-Natural-environment-2368.aspx)^22^. To account for the relative biological effectiveness of the different types of radiation, we applied the following weighting factors: 10 for α-radiation, 3 for low-β radiation (E < 10 keV), and 1 for other β-radiation and γ-radiation^36^. For each tree frog we calculate total individual absorbed dose rate by summing internal and external dose rates.

### Statistical analyses

All statistical analyses were conducted in R software (version 3.6.1, R Development Core Team, https://www.r-project.org/). We log transformed data of all parameters once we added 0.1 unit to each value of ^90^Sr and ^137^Cs dose rates, and to ambient, internal, external, and total absorbed dose rate (to avoid zero values impeding log transformations and generating problems with correlation analyses). Using the whole dataset, we conducted mixed-model regressions (lmer function, package lme4 version 1.1–23) to check for the relationships between ambient and total absorbed dose rate. In samples collected within localities with ambient dose rate > 1 µS/h, we conducted a mixed-model regression between internal and external dose rate. All regressions included the factor “locality” as random factor. We also conducted linear models to check for differences between localities in total absorbed dose rate, and internal-to-external ratio. For data plotting and visualization, we used the function ggplot included in the package ggplot2 (version 3.3.0).

## Supplementary Information

Supplementary Information 1.

Supplementary Figure S1.

Supplementary Figure S2.

Supplementary Figure S3.
