# Supplementary material for: Assessment of exposure to ionizing radiation in Chernobyl tree frogs (Hyla orientalis)
Source: Sci Rep. 2021 Oct 15;11:20509. doi: 10.1038/s41598-021-00125-9 (PMC8519934; doi:10.1038/s41598-021-00125-9)
Supplement: Supplementary file 1 — Supplementary Information 1. [file 41598_2021_125_MOESM1_ESM.docx]

**Figure S1.** Correlation between internal dose rate (μGy/h) and external dose rate (μSv/h) in breeding Eastern tree frog (*Hyla orientalis*) males sampled within Chernobyl Exclusion Zone.

**Figure S2.** Average contribution of ^90^Sr and ^137^Cs dose rate to the internal dose rate of breeding Eastern tree frog (*Hyla orientalis*) males sampled within the Chernobyl Exclusion Zone in localities with ambient dose rate > 1 μSv/h.

**Figure S3.** Average contribution of ^90^Sr and ^137^Cs dose rate to the external dose rate of breeding Eastern tree frog (*Hyla orientalis*) males sampled within the Chernobyl Exclusion Zone in localities with ambient dose rate > 1 μSv/h.
