# Supplementary figures and images for: Assessment of exposure to ionizing radiation in Chernobyl tree frogs (Hyla orientalis)
Source: Sci Rep. 2021 Oct 15;11:20509. doi: 10.1038/s41598-021-00125-9 (PMC8519934; doi:10.1038/s41598-021-00125-9)

Figure S1

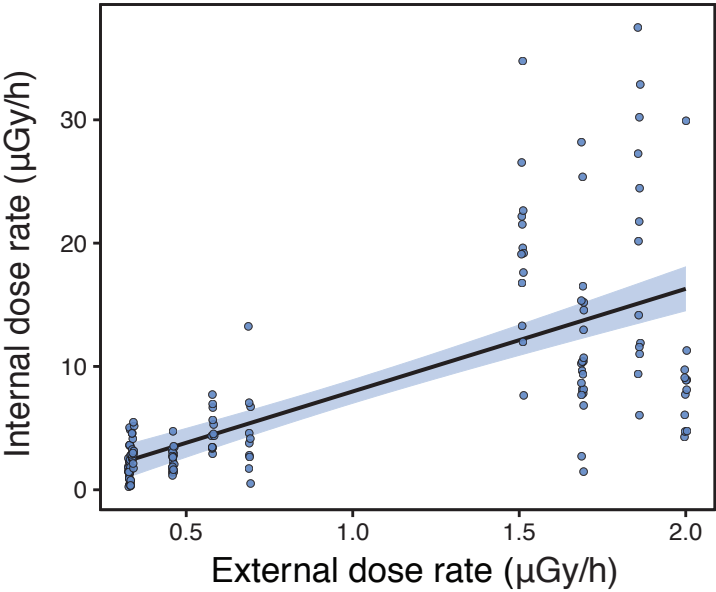

Supplement: Supplementary file 2 — Supplementary Figure S1. [file 41598_2021_125_MOESM2_ESM.pdf]

Figure S2

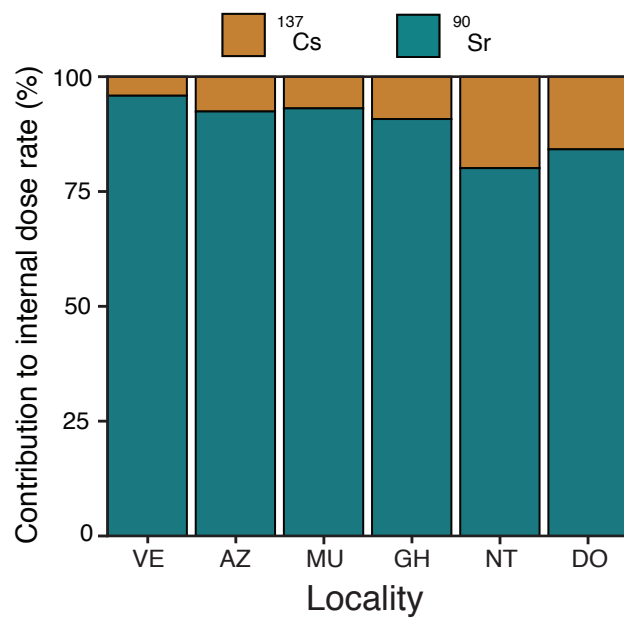

Supplement: Supplementary file 3 — Supplementary Figure S2. [file 41598_2021_125_MOESM3_ESM.pdf]

Figure S3

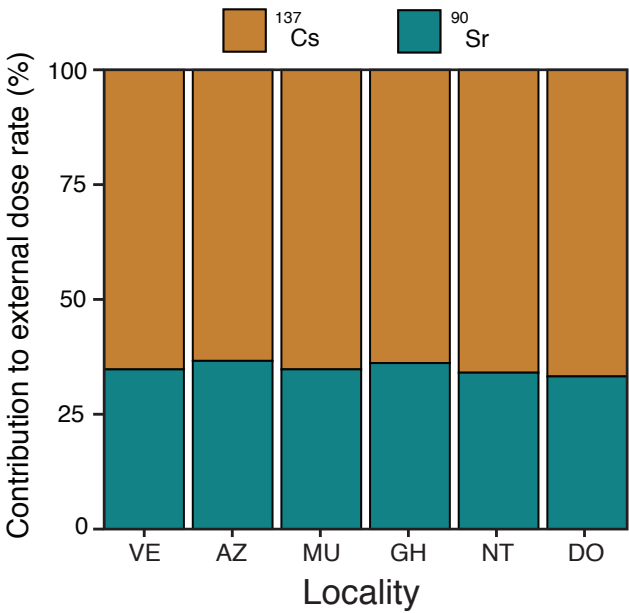

Supplement: Supplementary file 4 — Supplementary Figure S3. [file 41598_2021_125_MOESM4_ESM.pdf]
